# Design, development, and evaluation of a virtual reality game-based application to support computational thinking

**DOI:** 10.1007/s11423-022-10161-5

**Published:** 2022-10-26

**Authors:** Friday Joseph Agbo, Solomon Sunday Oyelere, Jarkko Suhonen, Markku Tukiainen

**Affiliations:** 1 School of Computing, University of Eastern Finland, P.O. Box 111, N80101 Joensuu, Finland; 2 School of Computing and Data Science, Willamette University, Salem, OR 97301 USA; 3 Department of Computer Science, Electrical and Space Engineering, Luleå University of Technology, 97187 Luleå, Sweden

**Keywords:** Computational thinking, Virtual reality, Immersion, Smart learning, Educational mini games, Nigeria

## Abstract

Computational thinking (CT) has become an essential skill nowadays. For young students, CT competency is required to prepare them for future jobs. This competency can facilitate students’ understanding of programming knowledge which has been a challenge for many novices pursuing a computer science degree. This study focuses on designing and implementing a virtual reality (VR) game-based application (iThinkSmart) to support CT knowledge. The study followed the design science research methodology to design, implement, and evaluate the first prototype of the VR application. An initial evaluation of the prototype was conducted with 47 computer science students from a Nigerian university who voluntarily participated in an experimental process. To determine what works and what needs to be improved in the iThinkSmart VR game-based application, two groups were randomly formed, consisting of the experimental (n = 21) and the control (n = 26) groups respectively. Our findings suggest that VR increases motivation and therefore increase students’ CT skills, which contribute to knowledge regarding the affordances of VR in education and particularly provide evidence on the use of visualization of CT concepts to facilitate programming education. Furthermore, the study revealed that immersion, interaction, and engagement in a VR educational application can promote students’ CT competency in higher education institutions (HEI). In addition, it was shown that students who played the iThinkSmart VR game-based application gained higher cognitive benefits, increased interest and attitude to learning CT concepts. Although further investigation is required in order to gain more insights into students learning process, this study made significant contributions in positioning CT in the HEI context and provides empirical evidence regarding the use of educational VR mini games to support students learning achievements.

## Introduction

Developing computational thinking (CT) skills such as problem-solving and recursive thinking is vital knowledge for computer science (CS) students in higher education institutions (HEI) (Settle, 2014). Critical thinking is required for a computational solution, and CT is necessary to equip young students for future challenges and employment needs (Agbo et al., 2019a, 2021a). According to the European Commission’s digital competence framework for citizens (DigComp 2.1), one of the eight proficiency levels required of every citizen is problem-solving skills (Carretero et al., 2017). This goal is laudable; however, the big question is whether such ambition is attainable in Africa where many of its countries are still struggling to introduce and maintain computer studies at secondary school levels (Mufeti & Sverdlik, 2017). Educational games and game-based learning (GBL) have been researched and identified as one way to present CT and problem-solving skills to young learners (Hooshyar et al., 2020; Lawrence, 2004; Seidel et al., 2019). Nowadays, educational games supported with immersive virtual reality (VR) technology have been explored to provide useful intervention for CS education (Bouali et al., 2019; Stigall & Sharma, 2018). In fact, a recent study demonstrated how immersive VR game-based applications could improve students’ common sense, creative thinking, and systematic reasoning (Segura et al., 2020), which are elements of CT. VR application provides immersion, presence, the immediacy of control, engagement, and interactions between learners and learning objects useful for enhancing learners’ cognitive benefits and reflective thinking (Lin et al., 2020). Cognitive benefits in the context of this study refer to abilities such as computational literacy, reasoning abilities, critical thinking, and memory gained from CT activities (Tsarava et al., 2019). According to Lin et al. (2020), the cognitive benefits that an educational intervention provides reveal its effectiveness to support learning and performance. Moreover, the characteristics of VR applications create an opportunity for students to experience smart learning (Agbo et al., 2019c, 2020). Although VR-based educational games for CT knowledge can engage students and motivate learning, research has shown that only about 12% of VR-based educational games are designed to support students in acquiring problem-solving skills (Radianti et al., 2020). Hence, more study is needed to provide an understanding of how VR-based educational games can enhance students’ learning in different contexts. At the moment, only one other published study by Segura et al., (2020) compared the effects of VR games on CT skills with a non-VR but equivalent game (Sukirman et al., 2022). Therefore, it is envisaged that the findings in this current study can help to validate and corroborate the findings from the Segura et al., (2020) study.

The relevance of CT and reasons why CT should be incorporated into the high school curriculum to facilitate problem-solving skills have been emphasized by previous studies (Lockwood & Mooney, 2018; Mannila et al., 2014). A systematic review of literature conducted by Lockwood and Mooney (2018) shows that scholars from developed countries have been integrating CT into the classrooms. However, in the case of developing regions such as Africa, there is little or no evidence of CT incorporation into the school curriculum. One recent study advocates for this feat where CT can be incorporated into the South African high school curriculum by conceptualizing a framework for CT in that context (Rothman et al., 2020). Another previous study developed a puzzle-based mobile learning system to facilitate students’ basic understanding of programming concepts and CT knowledge in Nigeria (Oyelere, 2018). These studies are part of the foundations necessary for CT knowledge, problem-solving skills, and programming education in the African context. However, in the HEI settings, many issues are yet to be addressed. For example, in Nigeria HEIs, challenges regarding increasing failure rate in undergraduate programming courses (Dasuki & Quaye, 2016; Sunday et al., 2020), the huge number of admitted students competing for limited learning resources (Agbo et al., 2019b), and the traditional lecture method of teaching by using the whiteboard, Powerpoint slides, and other multimedia (Oderinu et al., 2020), has lingered. These challenges create the opportunity for the design and development of an educational intervention that supports students’ CT knowledge, motivates students to learn programming concepts, and limits the pressure on the scanty resources by leveraging affordable technology that fosters learning such as smartphones. Even though some advanced VR headsets can be expensive, this study utilized Head Mounted Display (HMD) of less quality with low-cost that operates in the same way as Google cardboard, to make the application accessible to students (Biondi et al., 2016). Besides, the VR application in this study was developed with a free software package (Unity) and can be installed on students’ existing smart devices with the HMD, to make it viable. Furthermore, since CT is yet to be incorporated into computer science studies at the high school level in Nigeria, and the skill is required for programming education, it becomes necessary to develop a tool that facilitates students’ CT knowledge and problem-solving skills at HEIs. This educational intervention is important to prepare the young students for future job needs, otherwise, more generations of graduates will be produced by the HEIs without requisite CT skills.

Moreover, experts claim that students’ cognition can improve when they are involved in the process of ideation, creation, and implementation of an educational intervention that supports their own learning while gaining knowledge through experiential learning (Kolb, 2014; Lewis & Williams, 1994). Consequently, this study aims to demonstrate the design and implementation of the first prototype of an educational tool—iThinkSmart VR game-based application—co-designed with students to support their CT knowledge. The motivation behind the implementation of the iThinkSmart VR game-based application is the lack of interactive, engaging, and motivating ways of teaching CT concepts in Nigerian HEI. The need to develop students’ CT skills by associating game elements and VR features to develop educational VR game-based application has been proposed in a recent study (Sukirman et al., 2022). When students learn CT concepts through a theoretical approach, they can gain abstract knowledge that may not enhance their comprehension, hence a visualized approach to teaching CT concepts could be helpful to students. Therefore, this study leveraged experiential learning theory (Kolb, 2014) to co-design mini games with students and later refined those mini game concepts into a VR application as a step towards visualizing CT concepts that may fill the identified learning gap. This study including the development of the prototype was based on the design science research (DSR) methodology. The study examines how adding and using immersive VR 3D headsets to play mini games such as Tower of Hanoi game (when compared to playing the same game on a mobile phone’s 2D screen) affect student’s motivation and how that in turn affect students’ CT skills. In other words, the study investigates students’ CT competency after playing the iThinkSmart VR game-based application. Lastly, it examines students’ experience in terms of cognitive benefits, interest to learn CT, and attitude toward learning CT concepts after the experiment. Therefore, the study seeks to provide answers to the following research questions:

**RQ1**: To what extent does the iThinkSmart VR game-based application improve CS students’ computational thinking competency?

**RQ2**: Do CS students in a Nigerian university gain higher cognitive benefits of computational thinking from playing the iThinkSmart VR game-based application compared to those learning through non-VR game and traditional lecture method?

**RQ3**: Do the CS students learning computational thinking concepts through the iThinkSmart VR game-based application have higher learning interests and attitudes than those learning through non-VR game and traditional lecture method?

## Literature review

### Relevance of computational thinking

Scholars believed that CT had been around even before electronic computers surfaced with its origin traced to the 1950s (Tedre et al., 2018). With the arrival of interactive computers, Papert (1980) anticipated that its educational and societal values can influence human thinking and problem-solving. Indeed, history revealed that the term “computational thinking” was first used by Seymour Papert in his book entitled “Mindstorms” published in 1980. However, Wing’s re-introduction of the term about one and a half decades ago created an increasing interest in CT among scholars (Wing, 2008). In her view, Wing posits that CT influences every human endeavour since it is an approach to solving problems, therefore, “posing a new educational challenge for our society, especially for our children” (Wing, 2008 pp. 3717). An attempt to define CT can be challenging since a unanimous definition is not yet reached. Whether or not a precise definition is required in contrast to finding ways CT should be taught, including other nuances regarding what constitutes CT concepts remain a topic of discussion among scholars (Hu, 2011; DiSessa, 2018; Apiola & Sutinen, 2021). According to Aho (2012 pp. 832), CT refers to “thought processes involved in formulating problems so their solutions can be represented as computational steps and algorithms”. Following these definitions, significant discussions and studies on CT were carried out but majorly dwelt on K-12 and high school contexts (Lin & Shaer, 2016; Rothman et al., 2020). For example, Lin and Shaer (2016) demonstrated how a technology toy- (littleBits) can be explored as a learning tool for children to gain CT skills; Rothman et al., (2020) conducted a study on how to support CT in high schools through a conceptualized framework, which according to them, is capable of improving students’ CT skills. On the contrary, recent studies also show how CT is penetrating HEI (Agbo et al., 2019c; de Jong & Jeuring, 2020). According to scholars, the terms CT concepts (Lockwood & Mooney, 2018; Mannila et al., 2014) and CT knowledge (Hooshyar et al., 2021) are used interchangeably to mean the foundation topics in CT such as algorithmic thinking, problem decomposition, problem abstraction, pattern recognition, and recursive thinking. Therefore, this study refers to these topics whenever the term CT knowledge or CT concepts is used.

The issue of introducing young learners to CT concepts at different educational levels has been amplified elsewhere (Czerkawski & Lyman, 2015). For instance, Settle (2014) asserts that ways to support students “to learn recursion is clearly an open problem and should be addressed by computing education researchers.” (pp. 4). It is generally known that programming concepts such as recursion can be difficult for novice students to comprehend (Rojas-Salazar & Haahr, 2020), hence, a more practical approach must be designed to facilitate students' understanding of such complex concepts of programming in higher education level. Similarly, Mannila et al. (2014) had emphasized the importance of CT skills, which is why they should be incorporated into the educational curriculum at all levels. Although, there are still many differing views and opinions on whether CT is relevant, how it should be taught, and to whom (Apiola & Sutinen, 2021). However, a gradual penetration of CT into HEIs as shown by scholars justifies its relevance as a base knowledge to facilitate critical thinking and problem-solving skills (Agbo et al., 2019a; Apiola & Sutinen, 2021). Moreover, according to the European Commission’s DigComp 2.1 recommendation, digital competence and problem-solving skills are necessary not for the younger learners alone but for all citizens (Carretero et al., 2017). For developing countries such as Nigeria, scholars and educators require more effort to develop processes and educational interventions to foster CT knowledge among young learners.

### Development of computational thinking in Nigeria HEIs

Currently in Nigeria, the traditional lecture method where lecturers prepare and present their learning contents through the use of the whiteboard, Powerpoint presentation, and other multimedia remains the common practice in HEI (Oderinu et al., 2020). This traditional approach of teaching may limit students from gaining CT knowledge. For example, students are not motivated to learn abstract concepts such as algorithmic thinking, recursive thinking, problem abstraction, and decomposition through this traditional method (Dasuki & Quaye, 2016). Therefore, the need to provide an intervention to support students’ understanding of these abstract concepts is evident. One way to allow students to gain programming knowledge is through game-based approach as demonstrated by Lawrence (2004) where students learn data structure through games. Furthermore, research has shown the impact of visualization to teach programming concepts (Roberts, 2006)), and VR provides an opportunity for users to interact with learning objects in three-dimensional (3D) scenes in order to gain higher attention, motivation, and facilitates spatial cognition (Gomez, 2020; Kuznetcova et al., 2019). These days that educators are constrained with non-contact teaching due to pandemics or safety reasons, VR offers an excellent alternative for experimental learning and can replace conventional contexts (Cheng & Tsai, 2019). Aside from helping the students to gain more knowledge of programming through visualization and game-based approach, they are also able to gain and demonstrate CT competence required for nowadays employment in Science, Technology, Engineering, Arts, and Mathematics (STEAM) fields (Tedre & Denning, 2016).

The situation of CT in Nigeria HEI is such that not too many studies have been conducted to provide adequate CT skills to students. Among the few studies that focus on facilitating CT knowledge include Oyelere et al. (2018) who designed and developed a mobile learning application to teach elementary programming concepts by integrating Parsons programming puzzles into a traditional board game. Similarly, Oladipo and Ibrahim (2018) and Oladipo et al. (2017) have developed two interventions (CodeEazee and FULangS, respectively) to support students' understanding of CT concepts and build their problem-solving skills through self-learning. Another recent study implemented an online co-design of mini games with HEI students to facilitate their CT knowledge (Agbo et al., 2021a). All these studies related to CT knowledge in HEI in Nigeria have in some ways contributed toward developing CT skills among students. For example, Oladipo and Ibrahim (2018) reported that their tool (CodeEazee) allowed students who may have limited or no programming experience to design simple applications using the template, peer support, and gamification provided in the tool. Additionally, the evaluation of Oyelere et al. (2018)’s mobile learning application with students in Nigeria shows that the tool was viable and has the potential of supporting students in the Nigeria HEIs to gain basic programming knowledge and problem-solving through an indigenous game (Oyelere et al., 2019). It was also reported that students gained CT skills through the collaborative design of contextual mini games prototypes (Agbo et al., 2021a). Therefore, foundations for the integration of CT in HEIs are being created by these studies.

However, there is still a need to further develop interventions that mainly focus on the core concepts of CT, such as algorithmic thinking, problem decomposition, and recursive thinking. The current study does not only seek to contribute to the body of knowledge on CT but also explores ways to position CT studies in the Nigerian HEIs by supporting students’ knowledge through a VR game-based application.

### Virtual reality game-based learning for computational thinking knowledge

Scholars have researched educational games and game-based learning as one way to present CT and problem-solving skills to young learners (Lawrence, 2004; Seidel et al., 2019). Using game techniques to supplement the traditional teaching method can facilitate students’ understanding, enhance their learning experience, and improve their performance (Arrington et al., 2011). Hooshyar et al. (2021) shows that an adaptive computer game—Autothinking—has the potential of promoting students’ CT skills. Moreover, several studies have been conducted regarding the use of VR game-based applications in various educational disciplines (Kavanagh et al., 2017; Radianti et al., 2020). For example, Singh et al. (2021) developed a VR learning environment to support electronic engineering students’ laboratory experience. Their study experimentally evaluated students’ cognition and motivation for treatment and control groups and found that the intervention positively impact students’ understanding of electronic engineering topics taught in the VR environment for the experimental group compared to the control group. While some studies argue that VR game-based has a great deal of potential to support learning (Zhang et al., 2020), the aspects of its negative effect remain a topical issue (Pack et al., 2020).

In the computer science field, Arrington et al. (2011) designed and developed a VR game—Dr Chestr—with the goal to improve students’ performance and retention in computer science courses. The authors of Dr. Chestr VR game believe that the intervention can supplement the teaching of computer science topics and improve students' memorization. In another study, Bolivar et al. (2019) developed a VR game-based intervention to support players to gain CS concepts. The game mimicked the “Escape Room” scenario where a player could make decisions that depict an everyday situation. According to Bolivar and colleagues, the VR game has the potential of sparking CS interest in the player by providing simple games in an inviting mode for the players’ enjoyment while unconsciously learning CS concepts such as loop, arrays, and control structure. In addition, Bouali et al. (2019) demonstrated how VR game-based techniques can support students in learning object-oriented programming (OOP) concepts. The game was designed to allow players to visualize OOP concepts such as object creation, instantiation, and manipulations. Furthermore, Segura et al. (2020) developed a VR application called VR‐OCKS to support CS and engineering students to learn basic programming control structures such as iteration and conditional selection by controlling a humanoid player character within a VR environment. According to Segura et al. (2020), the VR game was found to support creative thinking and logical reasoning. Other disciplines aside from computer science are witnessing increasing growth of VR game-based applications to provide learning strategies. For example, Butt et al. (2018) developed a VR game-based application to aid nursing students to retain basic topics and nursing terms while taking care of patients. According to Butt et al. (2018), the VR game can transform nursing education from the traditional teaching method to a more interactive and motivating approach.

Aside from the motivation and engagement that VR game-based application provides to learners as reported in the literature (Bolivar et al., 2019; Bouali et al., 2019; Butt et al., 2018), other affordances of VR in education is cognitive benefits, which include memorization of concepts, critical thinking, and reasoning (Dalgarno & Lee, 2010; Segura et al., 2020). Currently, evidence-based studies on the affordances of VR in education are still limited (Zheng et al., 2018), hence, more research is required to broaden the knowledge domain which can, in turn, contribute to mainstreaming VR in education.

## The design science research methodology

In information system and engineering research, several design-oriented methods, including design experiments (Cobb et al., 2003), action research (Stringer, 2008), design thinking (Razzouk & Shute, 2012), and design science research (Hevner & Chatterjee, 2010) are popular. In the field of educational technology, the design-oriented methods emphasize the design, creation, and evaluation of a technology-mediated educational tool, pedagogical design, digital learning materials, etc., as integral part of the research process. However, one fundamental difference between these methods is the research procedures and frameworks defined to achieve the research goal. Design science research (DSR) is a method that involves a process for designing and creating solutions that address problems emanating from real-life situations (Oyelere, 2018; Apiola & Sutinen, 2021). As stated by Tommelein (2020), DSR is about “*designing and making artefacts to fulfill a purpose, and then testing and validating that they indeed are fit-for-purpose*” (pp. 340). DSR methodology has become popular nowadays in studies that center around innovations and information systems in order to bridge the gap between outcomes of academic research and actual industrial practices (Holopainen et al., 2020). According to Venable (2006), DSR stems from engineering and other applied sciences, and it is conceived by Herbert Simon in the mid-1990s. Since then, DSR has been widely used in different disciplines. In the field of CS education, Naidoo et al. (2012) showed how DSR could contribute to innovations and rigor of computing artefacts. Consequently, computer science scholars have been conducting research to either produce artefacts to solve practical problems using the DSR methodology (Benfell, 2020; Leinonen et al., 2016; Oyelere et al., 2018) or to design a theoretical framework that supports the use of DSR in the field (Elragal et al., 2019; Carstensen & Bernhard, 2019; Apiola & Sutinen, 2021). For example, Leinonen et al. (2016) in collaboration with the students developed mobile applications to foster students’ reflection during classroom learning by using the DSR method. According to Leinonen and colleagues, the study has the potential of fostering the practice of reflection. Recently, the work of Apiola and Sutinen which dwelt on exploring DSR to develop a framework for learning engineering and CT also underscores the relevance of DRS in CS education. Hence, our choice of DSR as the methodological approach in this study has been motivated by these pieces of evidence in the literature.

In the study conducted by Johannesson and Perjons (2014), DSR methodology was presented explicitly consisting of five interconnected activities. According to Johannesson and Perjons (2014), DSR provides an opportunity for researchers to structure their works in order to obtain a quality result. Therefore, the five specific DSR activities presented by Johannesson and Perjons towards designing artefacts include (i) problem explication, (ii) requirement definition (iii) design and development of artefact (iv) demonstration of artefact (v) evaluation of artefact. These activities can be executed iteratively or even linearly (Apiola & Sutinen, 2021), depending on how the intervention produced from the DRS method is verified to solve or meet the expectations delineated in the study (Brocke & Maedche, 2019).

Moreover, in the *problem explication* stage, the major activity is to examine what practical problem the DSR method must solve, whether the problem is significant in terms of impact and if it is well formulated and motivated to address a real-world situation (Johannesson & Perjons, 2014). The *requirement definition* stage of the DSR outlines a set of solutions in terms of what needs to be done in order to provide a solution to the explicated problem. The *design and development* stage of the DSR aims to create an artefact that is measured against the set of requirements in order to solve the explicated problem. The activities for the *demonstration of artefact* entail testing the developed artefact to prove whether it serves the purpose or can solve an instance of the problem explicated (Johannesson & Perjons, 2014; Tommelein, 2020). The *evaluation of the artefact* stage involves testing with the stakeholders to determine the extent to which the developed artefact can solve the problem explicated. In each of the DSR activity stages, a mixed-method approach can be used by researchers to execute the activities.

In a DSR project, an artefact and models or principles are mainly the outputs that contribute to the knowledge base (Johannesson & Perjons, 2014; Oyelere, 2018). Besides, the output from one phase can serve as the input for the next phase. The DSR activities presented by Johannesson and Perjons (2014) were followed in the development of the iThinkSmart VR game-based application. While this study focuses on the evaluation of the prototype developed, our previous studies have demonstrated other phases and activities of the DSR (Agbo et al., 2019a, 2019b, 2020, 2021a). “Design science research process of iThinkSmart VR game-based application” Section provides a highlight of how each phase of the DSR was followed up to the implementation phase of the iThinkSmart prototype.

### Participatory design and online co-design process embedded in design science research

Participatory design through a co-design process empowers users to practically contribute to the development of a technology-enabled intervention that fits their needs. According to DiSalvo et al. (2017), participatory design is a field of research that would not only allow stakeholders to contribute to the design process of a solution but also help to conceptualize and create the intended solution in order to better serve the stakeholders. Co-designing educational games with stakeholders is a viable approach to creating products that can directly impact learners in terms of addressing their specific learning needs (Loos et al., 2019). According to Seidel et al. (2019), developing educational game-based learning tools through a co-design process can create motivation among learners in order to gain cognitive benefits. This approach can mediate a lack of understanding of abstract learning contents such as recursion. In this current study, participatory design through an online co-design process was embedded into DSR methodology as part of the key instruments for implementing the VR game-based application to support students’ CT knowledge.

**Fig. 1:**
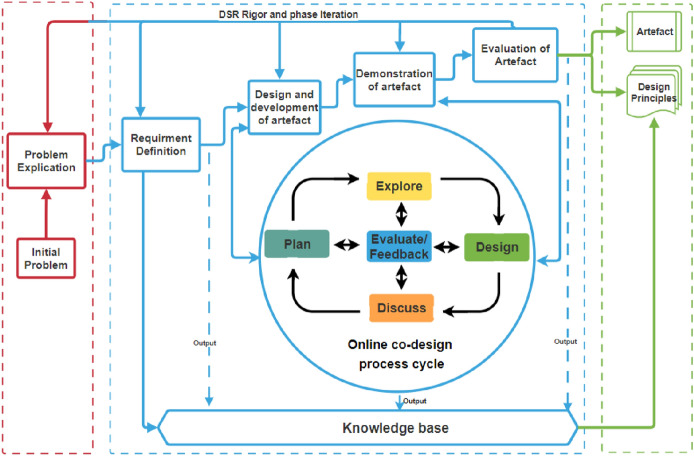
Co-design process embedded in design science research methodology (adapted from Agbo, 2022)

According to Agbo et al., (2021a, 2021b), online co-design involves the process of engaging users and researchers to conceptualize, create, and prototype an artefact through an online environment. The online co-design process strengthens the DSR methodology (Suero Montero & Kapinga, 2019) by facilitating activities three and four of the DSR phases. In other words, the participatory online co-design process is the driver to achieving activities in the design, development, and demonstration phases of the artefact. As presented in Fig. 1, the online co-design process cycle involves planning, exploring, designing, discussing, and evaluating stages.

These stages are also iterative, which provides more research rigor within the DSR methodology (Apiola & Sutinen, 2021). Although the co-design process to develop contextual artefacts has yielded useful output as acknowledged by scholars (Loos et al., 2019; Suero Montero & Kapinga, 2019; Seidel et al., 2019), the majority of the co-design process happens in a face-to-face mode. However, the current Covid-19 pandemic has constrained researchers to implement an alternative method of a co-design process with stakeholders. In this study, online co-design was adopted to suit the situation where a face-to-face meeting between participants and the researchers was not feasible.

### Design science research process of iThinkSmart VR game-based application

**Fig. 2:**
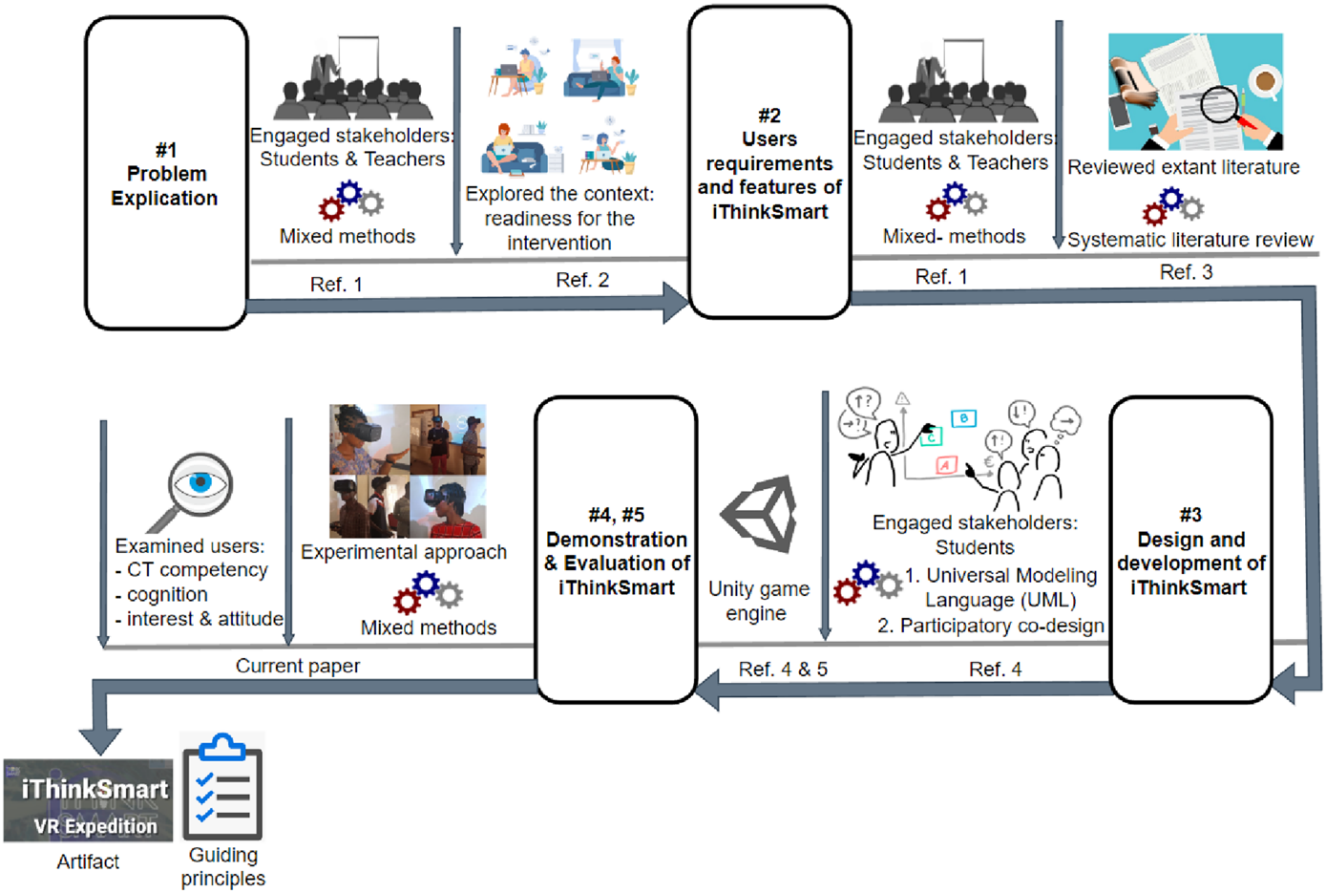
Overview of DSR activities to design iThinkSmart VR game-based application (*Ref. 1* = Agbo et al., 2019b*; Ref. 2* = Agbo et al., 2019c*; Ref. 3* = Agbo et al., 2019a*; Ref. 4* = Agbo et al., 2021a*; Ref. 5* = Agbo et al., 2021b; Agbo, 2022)

This section discusses the design and development of the first prototype of the iThinkSmart VR game-based application following the DSR phases of activities earlier presented in the beginning of “The design science research methodology”Section. An overview of how DSR activities were exploited to design and develop iThinkSmart VR game-based application is presented in Fig. 2.

#### Explicating problems behind the development of iThinkSmart VR game-based application

The problem explication stage of the DSR explored both interview and questionnaire approaches to engage the stakeholders (teachers and students) for the purpose of examining concrete problems surrounding the teaching and learning of CT and programming concepts in HEI in Nigeria. Besides, our earlier studies specifically investigated Nigeria HEI students’ readiness for the implementation of a smart learning environment to support CT skills and programming education.

#### Outlining features and defining requirements for iThinkSmart VR game-based application

In order to design and implement iThinkSmart VR game-based application, primary and secondary data gathered from students and published articles respectively were analyzed in two studies to gain insight into what exactly should the artefact do and how should it be developed to provide a useful outcome. The outcome from these two studies led to the outlining, refinement, and definition of requirements for the iThinkSmart VR game-based application.

#### Design and development of iThinkSmart VR game-based application

In designing the iThinkSmart prototype, we leveraged VR technology and a game-based approach as the media to facilitate learning activities. Although the discussion on the effectiveness of using a VR environment to facilitate learning of concrete topics has been on for over two decades (Javidi, 1999), recent studies have shown the potential of using immersive VR in teaching and learning in various settings (Çakiroğlu & Gökoğlu, 2019; Chen et al., 2020a, 2020b).

The Unity game architecture of model-view-controller-service (uMVCS) was followed (Rivello, 2020). MVCS is a high-level architecture with industrial standards adapted in Unity (Zheng et al., 2019). Unity is currently one of the most popular game engines used by both amateur and professional game developers around the world (Ferrone, 2019). In addition, Unity is a free software and allows for the deployment of VR applications on different platforms such as PCs, consoles, and mobile devices (Segura et al., 2020). In our implementation, a simple VR Google Cardboard or relatively simple HMD of about 5 US Dollars can be used to make the application affordable for students (Agbo et al., 2021a). After initiating the VR game, players' learning log data are instantiated and constantly updated on the database throughout the gameplay session. In addition, the iThinkSmart VR game computes the learners’ CT competency by applying Chaichumpa and Temdee’s (2018) objective distance (OD) model. The OD model measures the player's competence by applying the formula presented in Eq. 1 (Learning objective distance model (adapted from Chaichumpa and Temdee (2018))).

1 $$ OD_{i} \, = \,\left( {\frac{{S_{i} - C_{i} }}{{T_{i} }}} \right)\left( {\sqrt {\left( {C_{i} - S_{i} } \right)^{2} } } \right) $$

where, *OD*_*i*_ = Objective distance for an instance *i* of a learning object; *S*_*i*_ = Satisfactory score for an instance *i* of a learning object;*C*_*i*_ = Current score for an instance *i* of a learning object; and.*T*_*i*_ = Total score for an instance *i* of a learning object; *i* = 1,2, 3,…

The OD model proposed by Chaichumpa and Temdee (2018) was used to evaluate students’ competency while learning in a smart learning environment to enhance digital literacy (Temdee, 2021). OD measures the distance between the expected/satisfactory learner’s score ($${S}_{i})$$ and the current learner’s score ($${C}_{i}$$), mainly used to provide personalized support and intelligent feedback to a learner.

### Demonstrating iThinkSmart VR game-based application

As earlier explained, each of the mini games developed in the iThinkSmart VR game-based application is specifically targeted at using visualization to teach units of learning objects in CT concepts. Visualization of introductory programming concepts has been pursued by computer science educators for over two decades (Bergin et al., 1998). This study reinforces the use of visualization in enhancing students learning experience towards building their problem-solving skills, which will, in turn, improve their programming skills. The mini games contained in iThinkSmart application include (i) River Crossing, (ii) Mount Patti Treasure Hunt (MoPaTH), and (iii) Tower of Hanoi.

#### River crossing mini game

**Fig. 3:**
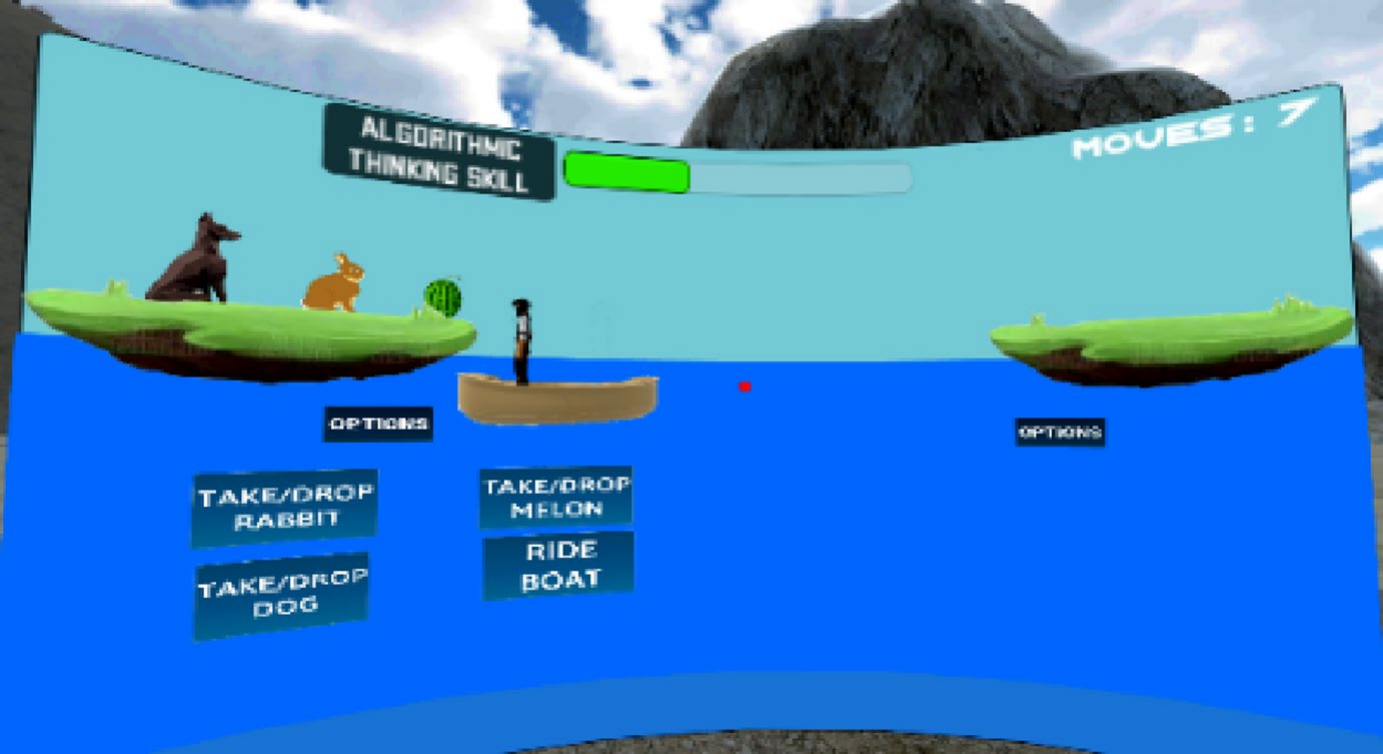
River crossing mini game

The river crossing problem is a known puzzle that teaches problem-solving in mathematics, CS, and engineering fields, majorly related to artificial intelligence (AI) algorithms (Ito et al., 2015). According to Ratnadewi et al. (2018), River crossing puzzle can be used to demonstrate an AI approach to solving the Breadth-first search (BFS) algorithm. In addition, the concept of optimization in CS can be taught by using the River crossing puzzle that requires an optimal operation to arrive at the best solution. The River crossing problem implemented in the iThinkSmart VR game-based application as shown in Fig. 3 allows students to apply mathematical knowledge of combination, logic, and reverse engineering (Papert, 2000) to arrange the items for an optimal solution. As demonstrated by cognitive scholars, the River crossing puzzle can foster learning and cognition when played within a highly interactive environment (Valle-Tourangeau et al., 2013).

#### Mount Patti Treasure Hunt challenge

**Fig. 4:**
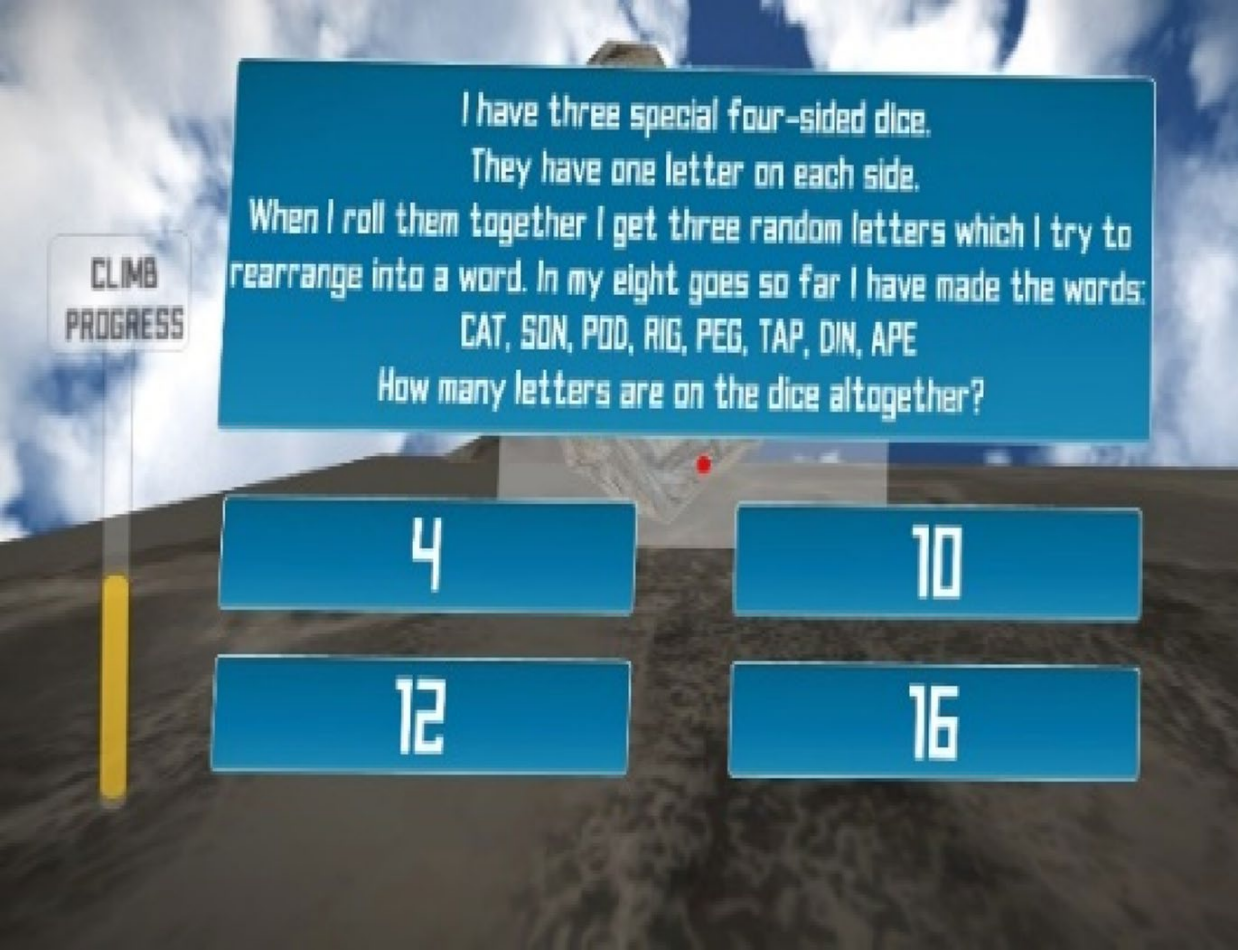
Mount Patti treasure hunt game

Mount Patti Treasure Hunt is a mini game conceptualized through a co-design process with students (Agbo et al., 2021a). Mount Patti is a popular mountain that is over 1500 feet tall and located in the north-central part of Nigeria. This mountain serves as a tourist site to many visitors and holds strong historical importance to the nation. For example, the country’s official name (Nigeria) was coined and named from the top of Mount Patti by the wife of a British governor in charge of the Nigeria Nothern protectorate in the 90’s. Students gamified the climbing of this mountain in order to unlock its treasures by playing puzzles consisting of CT problems as shown in Fig. 4. Players can only make progress to climb to the top of the mountain by answering correctly and timely puzzle questions that require critical thinking. The player is rewarded in points for any correct answer provided.

#### Tower of Hanoi challenge

**Fig. 5:**
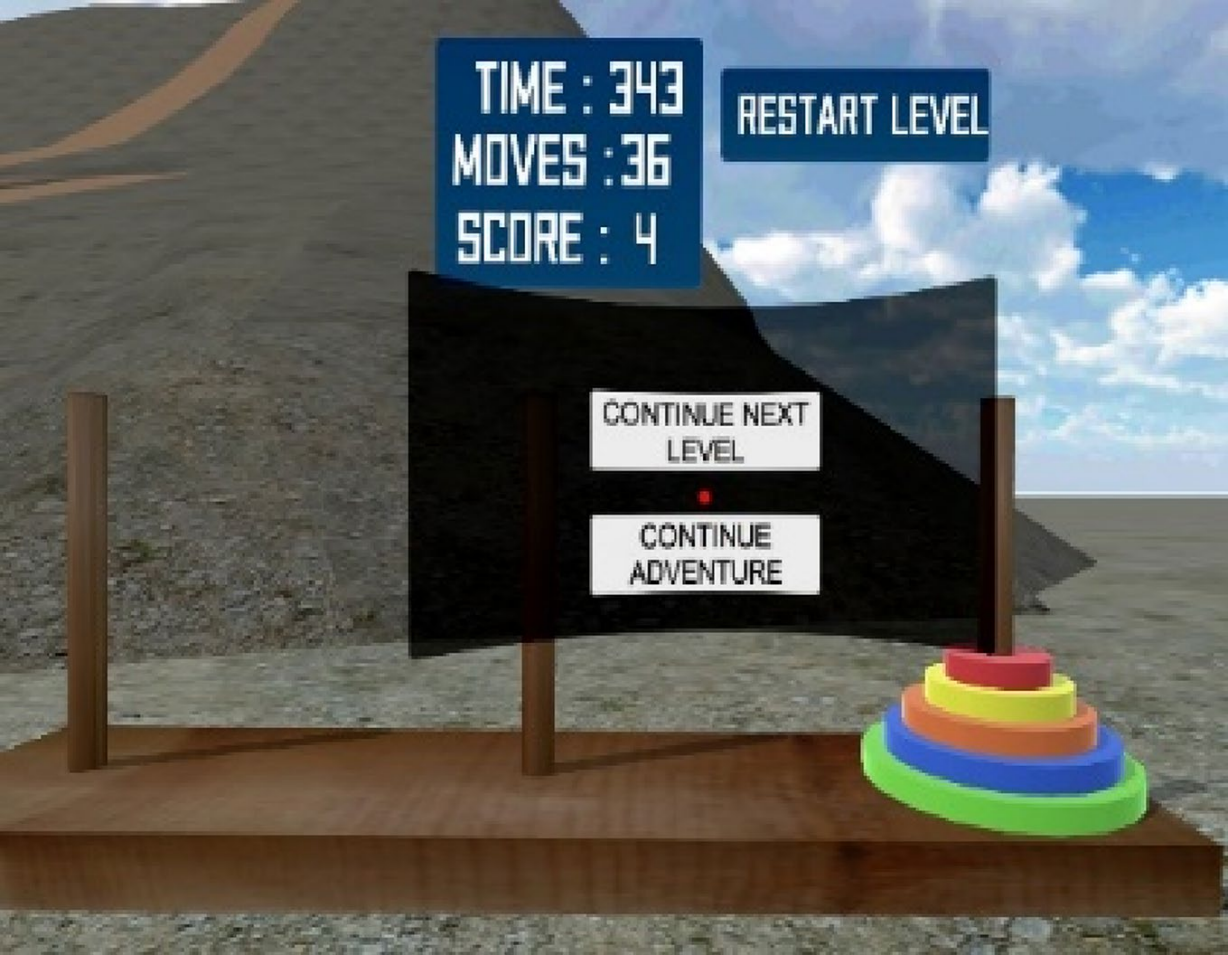
Tower of Hanoi game

Teaching recursion to CS students by using a mathematical function such as factorial and Fibonacci number can be challenging as students may not be able to build a mental model of the concept through these methods. According to Butgereit (2016), using games such as the *Tower of Hanoi*, a *Traveling Salesman*, or a *Road Inspector* can enhance memory comprehension of the recursion concept. A recent study by Rojas-Salazar and Haahr (2020) revealed how game-based approaches have been used to teach different concepts of programming including data structure and recursion. In addition, Roberts (2006) shows how the Tower of Hanoi mini game can enhance students’ understanding of recursion through the visualization of the concepts to facilitate interaction in a Java tutorial class. Similarly, Zhang et al. (2014) demonstrated how a game-based approach facilitates students’ understanding of recursion as a problem-solving technique. Their application—Recursive Runner—teaches students about the flow of execution of a recursive function through visualization in an introductory programming class. Hence, the iThinkSmart VR game-based application integrated the Tower of Hanoi shown in Fig. 5 as one of the mini games adapted to facilitate students’ CT skills through visualization while experiencing the gameplay in a VR environment.

Table 1 provides information regarding each mini game and what CT knowledge they are targeted to provide support for the students to gain.

**Table 1:** CT knowledge connected to iThinkSmart VR game-based application

Adapted techniques as mini games for CT knowledge	CT knowledge targeted				
	Algorithmic thinking	Recursive thinking	Problem decomposition	Problem abstraction	Pattern recognition
River crossing	✓	✓	✓	✓	✓
MoPaTH	✓		✓	✓	
Tower of Hanoi	✓	✓	✓	✓	✓

## Evaluation of iThinkSmart VR game-based application

The initial evaluation of iThinkSmart VR mini games was conducted in a north-central university in Nigeria. In order to evaluate students’ learning achievement in terms of CT competency, a pre-and post-test was conducted while questionnaires were also administered to collect quantitative data regarding the students’ attitude and interest to learn CT using the tool, and the cognitive benefits they gained from playing the mini games.

### Experimental procedure and participants

**Fig. 6:**
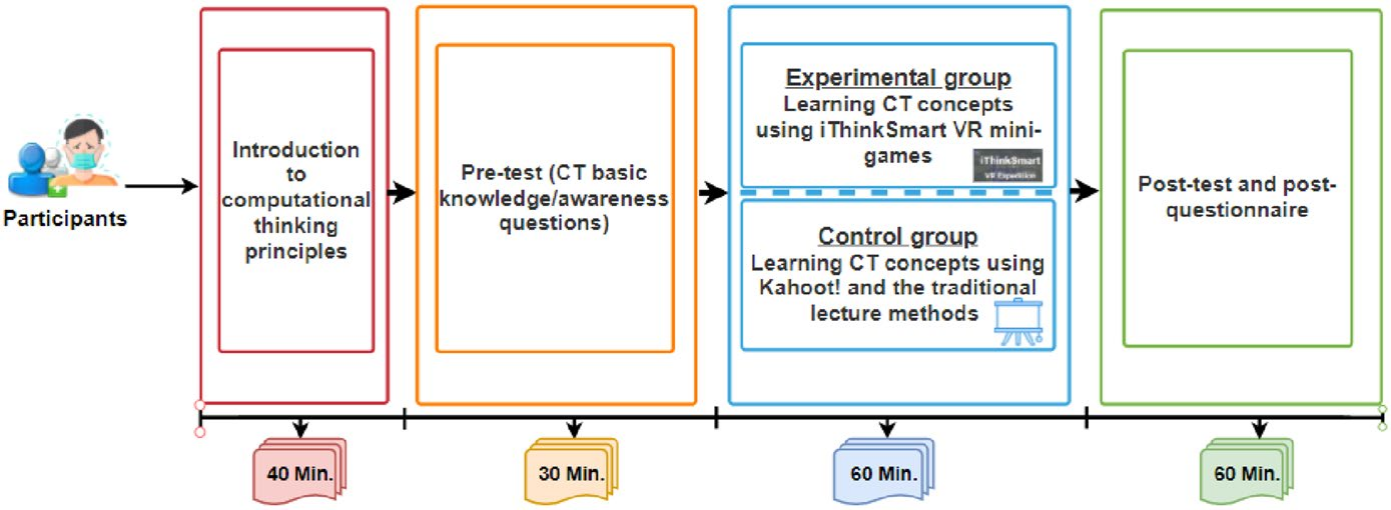
Experimental procedure

After receiving approval from the institutional head of a federal university located in the north-central region of Nigeria, purposive convenience samples of 47 CS students were recruited to participate in the study. Inclusion into the study was intentionally limited to CS students because they had previously completed CS courses that gave them sufficient background in CT and problem-solving. To validate this claim, the pre-test was conducted to mainly detect that all students who participated in the study had an equivalent basic knowledge of CT or demonstrate basic CT skills. Although, the study could not recruit students that are homogenous in terms of age and academic level, however, the students have commonness in terms of studying the same degree course and in the same university. The participants gave their informed consent through an online recruitment form where we introduced the goal of the experiment, informed the students that participating in the study is voluntary and they can withdraw from participating at any stage of the study. In addition, the participants gave their consent to use data collected during the experiment, including the images, for research purposes. Besides, this study conforms to the ethical guidelines of the Finnish national board on research integrity (Kohonen et al., 2019). Out of 60 students who registered to participate in the study, 47 turned up on the date of the experiment. This number of students is logistically manageable and considered sufficient for an initial evaluation of a VR application that followed the DSR iterative process of system development (Butt et al., 2018). The procedure followed to conduct the experiment is presented in Fig. 6. Before and after participating in the experiment, we collected the pre-and post-test data of two groups (experimental and control groups). Students’ CT competency from the two groups was compared based on their post-test scores while their interest to learn CT, attitude to learning CT, and cognitive benefits of CT skills after the experiment were analyzed and compared. In addition, the post questionnaire contained a single input form to generally obtain participants’ feedback regarding their perception of the VR application.

First, the participants were introduced to the concept of smart learning environments and CT principles. This first stage took 40 min to complete. Next, a short pre-test was administered to the students for 30 min. The pre-test consists of “yes” or “no” and multi-choice questions, which mainly aim to evaluate whether the two groups of students had the equivalent and basic CT knowledge and have the ability to improve their CT competencies. In the next stage, two groups (experimental and control) were formed through random sampling to engage in a learning session for 60 min using different learning approaches to gain CT knowledge as shown in Table 1.

The experimental group solved CT puzzles facilitated through River crossing and MoPaTH mini games to gain CT knowledge while playing the iThinkSmart VR game-based application. Whereas the control group solved similar puzzles and quizzes to gain CT knowledge by playing an online game-based platform (Kahoot!^1^), which was blended with the traditional teaching method using a Powerpoint presentation with Multimedia. Kahoot! is an online game-based platform to facilitate blended learning through quizzes, discussions, or surveys (Holbrey, 2020). Both groups’ CT learning content and quizzes were identical as shown in Table 6. The CT concepts such as algorithmic thinking, recursive thinking, problem decomposition, abstraction, and pattern recognition were part of the learning goal.

During the experiment, the researchers tried to maintain a balanced treatment for the experimental and control groups. For example, we ensured that all post-test questions administered to both groups were identical and time-bound where participants have a maximum of 60 s to answer a quiz. Besides, each participant accessed the learning media through their smartphones. While the experimental group installed the VR application on their personal devices before mounting it on the HMD, the control group accessed the online platform on their personal devices through a web link. All devices must be connected to the internet to ensure an online real-time recording of learning logs. Although the experimental group was fully immersed in the VR environment during their experiment, the control group did not have the full immersion experience. However, none of the participants expressed dissatisfaction as they all understood the purpose of the study, which necessitated the deliberate approach used for the experiment.

### Data collection instruments

This study collected data from two main stages of the experiment. The first stage of the data collection was the pre-test data while the second stage was the post-test and post-questionnaires. The post-tests include twelve multiple-choice questions of the CT competency test for beginners (BCTt v.1) (Zapata-Cáceres et al., 2020). The rationale for adopting the BCTt for the post-test questions was that it provides an opportunity for the researcher to develop multiple-choice questions with appropriate tasks that objectively evaluate students' performance (Weintrop et al., 2021). As compared to other CT assessment strategies (Weintrop et al., 2021), a performance-based test is an approach to CT assessment that is gaining scholars’ interest recently (Irgens et al., 2020). Furthermore, the BCTt approach was recently adapted in a related study to assess students’ CT competency in an educational computer game (Hooshyar et al., 2020). Because the majority of the targeted students are novices and do not have prior programming experience, we adapted BCTt as the starting point to introduce CT concepts to them. In the case of this study, the test difficulty level of the BCTt increase as the player starts from the first question and progresses to the last question as shown in Table 6. For example, one of the advanced multiple-choice questions uses random outcomes from rolling dice to backward engineer what is contained on their faces. Each question is weighted and has a fixed time of sixty seconds to provide an answer. The post-test was administered to both experimental and control groups to ensure that they received the same treatment. For the experimental group, the student’s test scores were assessed and computed using the OD model as shown in Eq. 1. That is, the total weighted score and satisfactory score were defined in the system while the player’s current score is obtained during the gameplay. Hence, the participants in the experimental group had their CT competency assessed for each test. On the other hand, the post-test questions were hosted on the Kahoot game and were played by participants in the control group. Each test had the same total weighted score and satisfactory score defined for both groups. While the data about the players’ CT competency scores based on the OD model were collected in real-time during the gameplay for the experimental group, the data of participants in the control group were downloaded as comma-separated values (CSV) file from Kahoot! online system and were manually computed using the OD formula.

Furthermore, the post-questionnaire instrument to measure students’ cognitive benefits after playing the iThinkSmart VR game-based application for CT skills was adapted from Lin et al. (2020), while the validated instrument for students learning interests and attitudes adapted from Hwang and Chang (2011), was used for the post-questionnaire. All the items in the questionnaire consist of a 5-point Likert scale (1-strongly disagree; 5-strongly agree). These instruments are presented in Table 7. Additionally, free form questions were added to the questionnaire administered to the experimental group in order to allow students who experienced the game, to provide feedback from their perspectives. For example, the free form questions include “please share with us your experience while playing the iThinkSmart VR mini games?”; “type in the box your comments regarding what you want to be improved in the next version of the game”. The Cronbach’s alpha for the questionnaire adapted for cognitive benefit is 0.89, for the learning interest is 0.78, and for the learning, attitude is 0.81, respectively. This indicates that Cronbach’s alpha condition for internal consistency and reliability of the instrument is satisfied.

## Results

This section presents the findings from the experiment conducted to evaluate the effect of the iThinkSmart VR game-based application to support CT skills. The result is majorly structured into three subsections to address the research questions. First, the CT competency analysis was conducted based on the CT competency test as shown in Table 6. Second, the analysis of CT cognitive benefits, interest, and attitude to learning CT was based on the instruments in Table 7. Additionally, the first-level analysis of students’ perception of the VR application is also presented at the end of this section.

### CT competence

To evaluate the extent to which iThinkSmart VR game-based application improves CS students’ CT competency, we computed the post-test score from the experimental group (n = 21, who learned CT concepts through iThinkSmart) and the control group (n = 26, who learned CT concepts through the traditional lecture method). Their scores were analyzed and presented in Table 2. According to the independent t-test result in Table 2, the mean score (μ) for the experimental group is 8.81 while the mean score (μ) for the control group is 6.96, respectively. The 21 participants in the experimental group (μ = 8.81, σ = 2.70) compared to the 26 participants in the control group (μ = 6.96, σ = 2.36) demonstrated significantly better CT competency scores, *t*(45) = 2.51, p = 0.016.

**Table 2:** The independent t-test result of the post-test score of CT skills from the groups

Variable		N	μ	σ	Std. Error	F-value	df	P-value	t	d
Post-test	Experimental	21	8.81	2.70	0.59	0.053	45	0.016	2.51*	0.74
	Control	26	6.96	2.36	0.46					

In addition, the effect size d (i.e., the difference between the means, μ_1_- μ_2,_ divided by the standard deviation, σ, of either group) was computed to investigate the strength of the t-test result. Cohen defined “*d* = 0.2” to indicate “small” effect size; “*d* = 0.5” to represent “medium” effect size, and “*d* = 0.8” to mean “large” effect size (Cohen, 1988). In this study, the d value is 0.74 which indicates a nearly large effect size, implying that those that learned CT concepts through iThinkSmart VR game-based application gained more CT competency compared to those that learned CT concepts using the traditional lecture method. This finding demonstrates the effectiveness of iThinkSmart VR mini games as an educational intervention for teaching and learning CT concepts.

### CT cognitive benefits

The second research question seeks to examine whether students who played iThinskSmart VR mini games gained higher cognitive benefits from CT compared to those who learned CT concepts using the traditional lecture method. To address this question, data from the cognitive benefit post-questionnaire (Lin et al., 2020) mean scores were used.

The *t*-test result in Table 3 shows that the mean score for the experimental group is 4.48 while that of the control group is 4.00, respectively. Comparing the mean shows a statistically significant difference (t = 3.96, p < 0.05), implying that students in the experimental group who learned CT concepts by playing iThinkSmart VR mini games gained higher cognitive benefits compared to students in the control group who learned CT concepts through the traditional lecture method.

**Table 3:** The t-test post-questionnaire result for cognitive benefits of CT

Dimension		N	μ	σ	t
CT cognitive benefits	Experimental	21	4.48	0.42	3.96*
	Control	26	4.00	0.40	

^*^p < .05

### CT learning interest and attitude

Regarding students' interest and attitude to learning CT after the experiment, we used their post-questionnaire mean scores to analyze and compare the experimental and control groups. The result from this analysis is presented in Table 4. For learning interest, the mean scores for experimental and control groups were 4.46 and 4.03, respectively, whereas, for learning attitude, the mean scores for experimental and control groups were 4.42 and 3.92, respectively.

**Table 4:** The t-test post-questionnaire result for learning interest and attitude of CT

Dimension		N	μ	σ	t
CT learning interest	Experimental	21	4.46	0.42	3.82*
	Control	26	4.03	0.35	
CT learning attitude	Experimental	21	4.42	0.40	6.34*
	Control	26	3.93	0.34	

^*^p > .05

According to the results obtained in Table 4, it shows that the mean scores for the experimental group and control group regarding interest to learn CT concepts after completing the post-questionnaire were statistically significant, (t = 3.82, p < 0.05), suggesting that the students in the experimental group who played iThinkSmart VR mini games have a higher interest in learning CT concepts compared to the students that do not use the educational game to learn CT concepts. In addition, the result also suggests that students who played iThinkSmart mini games show a significantly higher CT learning attitude (t = 6.34, p < 0.05) compared to those who do not play iThinkSmart mini games.

### Analysis of participants’ experience with iThinkSmart VR game-based application

This section presents the analysis of participants’ experience with the iThinkSmart VR game-based application following Ge et al. (2021)’s approach to analyzing participants’ first-level reaction to the gameplay. Table 5 presents the themes obtained from the participant’s responses to the free form on their experience with iThinkSmart VR game-based application. Generally, the major feedback from participants was centered around the interaction and response to the game. Students seem to find it difficult to move freely in the virtual world. Another related comment touches on the aspect of allowing only walking mode in the virtual world and not running mode.

**Table 5:** Analysis of students’ responses regarding their experience of playing the VR mini games

Themes	Analysis of responses and aspects of the game that need improvement
Graphics and aesthetic	Participants gave their feedback regarding the graphics. Specifications on how the graphics should be improved to a certain value of frames per second (fps). Examples of responses include “The graphics of the game should step up to 720p and 60fps” “The graphics should be enhanced” “The graphics of the game need an enhancement there.”
Navigation, instructions, demo, and hints	Some of the responses from participants suggest the need to improve the navigations, instructions, and tutorials provided within the game to guide players Some examples of the responses read: “It will be better and easier to learn and navigate through the virtual environment if there was a kind of demo mode or detailed description/inscription on different objects and different locations on how to navigate and the available options to make” “An overview of how to play the game should be given so that anyone playing the game for the first time can easily play the game not necessarily attending a seminar on it or reaching out for a tutor” “Many games usually have a short demo, giving the steps and how the game should be played, iThinkSmart game should try and improve in this line…” “I want the arrow indicating the direction to be very visible….”
Movement, Speed, and Controller	Students anticipate an improvement in the aspect of speed, walking, and running behavious of the game, and easier navigation. For example, some participants assert “…speed of the avatar only has pace and walking mode but not running mode, and it hinders players who would like to break records by making the fastest gameplay time” “…the ability for the movement to be faster”
Support for device	There was feedback on how a device could not properly support the smooth operations of the game. The participant remarked “… it seems the VR application doesn’t run smoothly on all phones—some phones were not responding to movement”
Learning content	The participants wished to see more learning content deployed within the game. An example of a response reads “…more topics to be added in the game”
Satisfaction with learning outcome	A few participants praised the game and expressed satisfaction with how it provides CT knowledge and problem-solving skills. An example of a response reads: “…good job for developing such computational thinking system, I think it is very good in providing knowledge on problem-solving” Some of the participants were positive about the game and even expects the game to be hosted on the Google play store for easy download by students from anywhere in the world. For example, a participant’s response is: “…let the game be launched to play store so that the world can have experience of iThinkSmart mini games too”

While these responses are very useful for improving the VR application, they basically revealed that the first prototype was able to truly engage the students to experience a high-fidelity interaction with the mini games. However, detailed information on what, how, and why certain features are required is still needed. This kind of in-depth investigation with users can be done through interviews to provide more concrete information for the enhancement of the VR application.

## Discussion

This study demonstrates the design, development, and initial implementation of a smart educational VR game-based application (iThinkSmart) aimed at supporting students’ understanding of CT concepts and gaining CT competence. CT skill is essential for all citizens (Carretero et al., 2017). The educational mini game is one way to support students’ CT and problem-solving skills (Bolivar et al., 2019; Hooshyar et al., 2020). The iThinkSmart VR mini games were developed to provide players with immersion, a high level of interaction, and engagement in a virtual world. In addition, iThinkSmart VR game-based application was modeled to compute students’ CT competency using the OD model (Chaichumpa & Temdee, 2018) in real-time during the gameplay and the computed CT competency data were further used in the analysis. Unlike the approach used to determine CT competency in a previous study (Hooshyar et al., 2020), the method adopted in iThinksmart to measure CT competency helps to understand how students learn and apply CT skills in different situations during gameplay. Through this type of learning approach as provided by the iThinkSmart VR game-based application, students can gain more knowledge on CT concepts and build their CT skills (Segura et al., 2020). Therefore, we argue that assessment of CT competency should be integrated into the learning tool so that learners’ indicators such as activities performed to solve tasks, scores obtained, and other parameters of learning progress can be computed objectively in order to provide personalized feedback.

Aside from supporting students learning experience through game-based learning to gain CT skills as demonstrated by Hooshyar et al. (2020) and Hooshyar et al. (2021), this study suggests that students can be more immersed to interact with concrete objects that facilitate experiential learning (Kolb, 2014) of CT in a learning environment using virtual reality games compared to computer-based games. Furthermore, this study showcased the use of the DSR framework as a pragmatic approach and theoretical foundation for designing and developing iThinkSmart VR educational application to support the learning of CT concepts (Apiola & Sutinen, 2021).

To evaluate the efficacy of the VR application to support students’ CT skills compared to the traditional lecture method, we defined three research questions that this study addressed. Specifically, the study (i) examined the extent to which iThinkSmart VR game-based application helped the students to gain CT skills compared to the traditional lecture method, (ii) investigated whether iThinkSmart VR mini games provide higher cognitive benefits in terms of CT knowledge to the students compared to using another traditional lecture method, (iii) measured whether iThinkSmart mini games have an effect on students’ interest and attitude to learn concepts after playing the mini games. Previous studies have investigated similar parameters regarding the impact that educational games have on students’ CT knowledge and skills (Banic & Gamboa, 2019; Hooshyar et al., 2020; Parmar et al., 2016). Discussion of this study’s findings is structured according to the research questions.

### CT competence

The finding from this study shows that iThinkSmart VR game-based application improves students’ CT competence and problem-solving skills more than the traditional technology-enhanced learning approach. In order words, students who played the educational intervention (iThinkSmart VR mini games) demonstrated higher CT competency compared to students who do not play iThinkSmart VR mini games but learned CT concepts using a different approach. To be more specific, the mini games integrated into the iThinkSmart VR game-based application present better learning scenarios and goals such that students who played the games were able to resolve challenges on CT problems presented to the player within the virtual world. As apparent in this study, the traditional approach of teaching and learning CT concepts such as algorithmic thinking, problem decomposition, problem abstraction, pattern recognition, and recursive thinking even on 2D computer game could not provide sufficient CT skills such that most of the students in the control group could not obtain the optimal solution for resolving CT problems such as River crossing. One way to explain the reason for this result is the personalized experience and motivation that iThinkSmart VR (3D) game-based application provides (Dalgarno & Lee, 2010; Segura et al., 2020; Singh et al. 2021) where players are guided to interact with the learning objects, instructions, and timely feedback within a virtual world. This finding conforms to previous studies that have investigated VR environment for teaching engineering students (Singh et al. 2021) and other educational games for CT skills (Banic & Gamboa, 2019; Hooshyar et al., 2020). Furthermore, our finding corroborates the findings from similar study by Segura et al., (2020) who found that the use of VR-OCKS strengthened the spatial orientation and autonomy of the users, enhanced their common sense, creative thinking, and systematic reasoning. Unlike Segura et al. (2020)’s study that examined how VR application foster learners’ CT in K-12 setting, our study investigated how VR mini games facilitate computer science college novices’ CT skills. From the experimental point of view, our finding confirms that VR game-based application has a huge potential for teaching computer science and computational thinking concepts (Segura et al., 2020; Stigall & Sharma, 2018).

### CT cognitive benefits

Regarding the CT cognitive benefits gained by students from the experimental and control groups, our findings show that iThinkSmart mini games improved students' CT cognitive benefits in the experimental group than students who used the traditional lecture method in the control group. The cognitive benefits of VR educational tools have been measured against learning effectiveness (Lin et al., 2020). According to Lin and colleague, VR features such as immersion, interactivity, immediacy, aesthetics, and presentational fidelity are factors that influence cognitive benefits, which further have an effect on learning performance and effectiveness. We can argue that the finding from this study where students who played iThinkSmart VR mini games demonstrated higher cognitive benefits compared to students who do not play the games is not unconnected to Lin et al. (2020)’s finding. In other words, the VR features such as immersion, interactivity, immediacy, aesthetics, and presentational fidelity, which iThinkSmart VR game-based application provides to its players could have contributed to their higher cognitive benefits as revealed in earlier studies (Arrington et al., 2011; Dalgarno & Lee, 2010). In another study that showcased similar findings to this study, Chen and his colleagues revealed positive evidence that visualization and creation of disaster scenarios through VR enhance students’ comprehension, computational thinking, and understanding of artificial intelligence of things (AIoT) course (Chen et al., 2020a, 2020b). Similarly, Singh et al. (2021) found that a VR environment to teach electronic engineering support students' cognition compared to traditional teaching methods. Essentially, while the previous study by Lin and Shaer (2016) revealed that African young students were engaged in gaining CT competency through littleBits, this study explored how students in higher education in Africa can equally demonstrate CT competency.

### CT learning interest and attitude

This study also examined the effect of iThinkSmart VR mini games on students' interest to learn CT concepts and their attitude toward learning CT concepts by analyzing the mean score of the post-questionnaire filled by participants from the experimental and control groups after completing the experiment. The two groups were compared, and the result revealed that students who played iThinkSmart VR mini games are perceived to have both higher learning interests and attitudes. This finding suggests that iThinkSmart VR game-based application has the potential to serve as an effective learning tool for CT skills compared to conventional learning environments (for example Powerpoint based teaching approach). This finding conforms to previous studies that have investigated students’ learning interests and attitudes by using educational interventions (Hooshyar et al., 2020; Hwang & Chang, 2011; Oyelere et al., 2018). iThinkSmart VR game-based application presents CT concepts in a virtual world, which may cause learning to be more complex for novices in terms of immersion and interaction than the conventional computer or mobile-based educational games (Hooshyar et al., 2020; Oyelere et al., 2018). However, this approach provides useful reinforcement of learning concepts that can facilitate programming skills through visualization in the 3D environment (Dalgarno & Lee, 2010; Zheng et al., 2018). Besides, this study conforms to the findings of Bolivar et al. (2019) who developed a VR game-based intervention to support players to gain CS concepts with the intention to spark their interest in CS by learning concepts such as loop, arrays, and control structure.

Overall, the result from the experiment suggests that iThinkSmart VR game-based application, although in a prototype stage, has the potential of supporting students to gain CT competency by mediating the traditional approach of teaching and learning necessary for their future job. While this study seems to report a positive outcome, the opinion of the participants regarding elements of games suggests the need for improvement.

### Users’ perception and suggestions for system improvements

This study revealed several aspects that require improvement as perceived by participants who played the developed prototype. The thematic aspects as presented in the result section include graphics and aesthetics, navigations, speed, learning topics, and smooth operations on different kinds of devices. Regarding the graphics and aesthetics, the study revealed that users’ experience rates the application prototype not up to the expectation, which would require further improvement. The VR environment was modeled as a representation of a real-world object. Hence, the more users perceive the virtual world to be real, the better their experience (Zhang et al., 2020). Further, users expect the system to be more user-friendly by providing hints and demonstrations that can help novices to operate the mini games in a VR environment. Notably, most of the participants in this study were new to the VR environment, hence, this can affect their perception in terms of the user-friendliness of the VR application. Additionally, their perception of the learning goal of the mini games reflects that they are motivated to gain more knowledge using the game-based approach, which align with findings from Segura et al. (2020). Thus, users anticipate more mini games to be integrated into the system. As part of our future plan to improve the system, all points identified by users would be taken into consideration to form part of the requirements for the next iteration of the research.

### Study limitations and future research

This study witnessed a few limitations. First, the sample size used for the experiment is rather small. The reason for this small sample was because the study was conducted during the Covid-19 pandemic era where a large gathering of students was not recommended by the university. In addition, this study did not explore a qualitative approach by collecting data from participants through interviews, which may have provided more insight into their learning process and experience. Aside from the small sample and absence of a qualitative method, the experimental condition was limited to only one university and a discipline. Notwithstanding, the sample and setting used for this experiment are sufficient for an initial evaluation of the educational intervention. However, authors will utilize the opportunity provided by these limitations to explore future research. In other words, our future research will recruit more sample sizes and involve different experimental settings, universities, and disciplines in order to understand a broader phenomenon in terms of the effectiveness of the VR educational intervention. In the future, the authors will also conduct a rigorous quantitative and qualitative study to examine participants’ CT competency using other assessment strategies, learning processes, perceptions, and experiences. Meanwhile, the prototype would be further improved to take into account aspects that users have expressed their expectations.

## Conclusion

In this study, the authors have demonstrated how a VR game-based application (iThinkSmart) aimed at supporting CT skills through mini games was designed and implemented. The study followed the DSR methodology in a phase-wise process to design, implement, demonstrate, and evaluate the application. The evaluation of the VR application was conducted by recruiting CS students from a Nigerian university who voluntarily participated in an experimental process. In order to determine the efficacy of the iThinkSmart VR game-based application, the experiment was conducted consisting of two randomly selected groups. Precisely, the study investigated students’ CT competency, examined students’ cognitive benefits, interest to learn CT and attitude toward learning CT concepts after completing the experiment.

Our findings generally suggest that iThinkSmart VR game-based application promotes students’ CT competence, as the experimental group demonstrated more problem solving, algorithmic thinking, problem decomposition, abstraction, pattern recognition, and recursive thinking skills. These CT concepts were integrated into the adapted mini games for teaching CT through VR and also formed the contents for the traditional method used during the experiment. Regarding the other components of student learning achievement, the study revealed that students in the experimental group gained higher cognitive benefits than those in the control group. In addition, this VR educational application has shown to promote students’ interest to learn CT concepts and improve their attitude to learn CT concepts. By implication, this study provides insight for educational technologists and researchers regarding the strategy suitable for positioning CT in HIEs since mini games in a virtual environment have shown to be a suitable approach for CT knowledge in this context. The use of state-of-the-art technology to mediate learning is increasingly studied and this study also contributes to knowledge in terms of how VR and game-based learning can be harnessed to mediate learning. In addition, this study recommends that educational VR developers could utilize low-cost HMD with hand controllers and a smartphone device can provide viable media for advancing CT skills in the context of a developing country. Furthermore, designing a CT intervention that assesses students’ learning and competency through gameplay is an important element of a technology-enhanced learning environment and should be considered more seriously. In particular, the study concludes by remarking that educators should consider employing VR mini games in their CT classes to provide more effective learning achievements.

## Data Availability

Not applicable.

## Appendix

See Tables

**Table 6:** CT competency test

CT questions	Multiple choice	
Which of the following is an example of thinking computationally?	a.	Planning out your route when going to meet a friend
	b.	When going to meet a friend, wandering around until you find them
	c.	When going to meet a friend, asking a parent to plan your route for you
Which of the following is NOT an example of computational thinking?	a.	Letting the bossiest friend decide where you should all go
	b.	Considering the different options carefully before deciding upon the best one
	c.	Discussing with your friends how much time and money you have before choosing from a shortlist of places
What is a complex problem?	a.	A problem that, at first, is not easy to solve
	b.	A problem that, at first, is not easy to understand
	c.	A problem that, at first, is not easy to solve or to understand
Which of the following is NOT a computational thinking technique?	a.	Abstraction
	b.	Algorithms
	c.	Coding
Which computational thinking technique involves breaking a problem down into smaller parts?	a.	Decomposition
	b.	Abstraction
	c.	Algorithms
To create a successful computer program, how many computational thinking techniques are usually required?	a.	Two
	b.	Four
	c.	Three
When is a computer most likely to be used when using computational thinking?	a.	During decomposition
	b.	At the end, when programming a computer
	c.	When writing algorithms
Four adventurers (Alex, Brook, Chris and Dusty) need to cross a river in a small canoe. The canoe can only carry 100 kg. Alex weighs 90 kg, Brook weighs 80 kg, Chris weighs 60 kg and Dusty weighs 40 kg, and they have 20 kg of supplies. Select a possibility for getting across	a.	Brook and Dusty can row across, Dusty returns
	b.	Alex and Chris row across and Chris returns
	c.	Chris and Dusty row across, Dusty returns
I have three special four-sided dice. They have one letter on each side. When I roll them together, I get three random letters which I try to rearrange into a word. In my eight goes so far, I have made the words: CAT, SON, POD, RIG, PEG, TAP, DIN, APE. How many letters are on the dice altogether?	a.	10
	b.	12
	c.	16
There are five gears connected in a row, the first one is connected to the second one, the second one is connected to the third one, and so on. If the first gear is rotating clockwise what direction is the fifth gear turning?	a.	Clockwise
	b.	Anti-clockwise
	c.	Oscillatory-wise
You are about to leave for holiday, but you forgot socks! You race back to your room, but the power is off so you can’t see sock colours. Never mind, because you remember that in your drawer there are ten pairs of green socks, ten pairs of black socks, and eleven pairs of blue socks, but they are all mixed up. How many of your socks do you need to take before you can be sure to have at least one matching pair?	a.	Take three sucks, you are guaranteed of having a matching pair
	b.	Take four socks, you are guaranteed to have at least one matching pair
	c.	Take no sucks since the room is dark and no guarantee of having a matching pair
You must take two different tablets (A and B) each day. If you forget one, or take more than one of any type, you will get sick. Unfortunately, they look exactly the same, and to make matters worse, with only two days’ supply left, you drop all 4 tablets on the floor together! What can you do?	a.	Take half of each tablet today, and the rest half of each tablet tomorrow
	b.	Guess and take any two of the tablets today, and the remaining two tomorrow
	c.	Do not take any of the tablets and remain sick

6 and

**Table 7:** List of questionnaires adapted for the initial evaluation of the VR game-based application

Conctructs	Items
Learning interests	1. Computational thinking course is interesting
	2. Learning more about computational thinking concepts is interesting
	3. It is interesting to answer those computational thinking questions while learning through games
	4. I always look forward to taking computational thinking topics and prepare for programming class
	5. The teacher’s instructions on computational thinking concepts have attracted my attention
	6. Anything concerning computational thinking is always interesting to me
	7. The computational thinking course is more interesting to me in comparison with other courses
	8. Other courses do not attract me as much as the computational thinking course
Learning attitudes	1. The computational thinking course is worth studying
	2. It is worth learning things about computational thinking
	3. It is worth learning the computational thinking concepts well
	4. It is important to learn more about computational thinking concepts, such as algorithmic thinking, recursive thinking, and problem solving
	5. It is important to know and apply the computational thinking concepts
	6. I will actively search for more information and learn about computational thinking concept
	7. It is important for everyone to take the computational thinking course
Cognitive benefits	1. The game approach made the comprehension of computational thinking topic easier
	2. The game approach made the memorization of computational thinking topic easier
	3. The game approach helped me to better apply what was learned
	4. The game approach helped me to better analyze the problems
	5. The game approach helped me to have a better overview of the content learned

7.

## Footnotes

1 https://kahoot.com/.
